# The use of a visual motor test to identify lingering deficits in concussed collegiate athletes

**Published:** 2020-04-16

**Authors:** Katherine J. Hunzinger, Erik W. Sanders, Horace E. Deal, Jody L. Langdon, Kelsey M. Evans, Brandy A. Clouse, Barry A. Munkasy, Thomas A. Buckley

**Affiliations:** ^1^Department of Kinesiology and Applied Physiology, University of Delaware, Newark, Delaware; ^2^Interdisciplinary Biomechanics and Movement Science Program, University of Delaware, Newark, Delaware; ^3^Department of Athletics, Ohio Northern University, Ada, Ohio; ^4^Department of Intercollegiate and Georgia Southern University, Georgia; ^6^Department of Health Sciences and Kinesiology, Georgia Southern University, Georgia; ^5^Department of Vision Source Signature Eye Care, Statesboro, Georgia; ^7^Department of The Brody School of Medicine, East Carolina University, Greenville, North Carolina, United States

**Keywords:** mild traumatic brain injury, Dynavision, reaction time, coordination

## Abstract

**Background:**

Emerging evidence suggests neurophysiological deficits, such as visual motor coordination (VMC), may persist beyond clinical concussion recovery. Instrumented measurement of upper-limb VMC is critical for neurological evaluation post-concussion and may identify persistent deficits further elucidating persistent neurophysiological impairments not detected by the current clinical assessment battery.

**Aim:**

The aim of the study was to determine if a VMC test identifies persistent deficits in concussed collegiate student-athletes who have returned to baseline on clinical concussion assessments.

**Methods:**

Thirteen recently concussed intercollegiate student-athletes (male: 7, 18.9±0.7 years, 175.5±12.4 cm, 75.5±23.2 kg), and 13 matched control student-athletes (male: 7, 19.3±1.1 years, 173.5±11.9 cm, 75.8±19.9 kg) completed two testing sessions (T1: <48 h after clinical recovery; T2: 30 days post-concussion) on a visual motor exam. The outcome measures were A* Average score (average number of lights hit on A* exam), simple visual reaction time (SVRT)-RT, and movement time (SVRT-MT) on the Dynavision D2. The dependent variables were compared with a 2 (group) × 2 (time) repeated measures ANOVAs.

**Results:**

There was no group interaction in A* average score (*F*(1,24)=0.036, *P*=0.849), SVRT-RT (*F*(1,22)=0.319, *P*=0.575), and SVRT-MT (*F*(1,22)=1.179, *P*=0.188). There was a main effect for time on A* average score (T1: 76.3±10.4 hits; T2: 82.7±11.2 hits; *F*(1,24)=38.1, *P*≤0.001) and SVRT-RT (T1: 0.31±0.04; T2: 0.29±0.04 s; *F*(1,22)=4.9, *P*=0.039). There was no main effect for SVRT-MT. There were no group differences at either time point.

**Conclusions:**

Among recently concussed collegiate student-athletes, no persistent deficits were identified in VMC beyond clinical recovery when assessed by Dynavision D2. This VMC exam may not provide a useful means of tracking recovery following concussion likely due to a substantial practice effect.

**Relevance for patients:**

While post-concussion neurophysiological deficits persist beyond clinical recovery, the laboratory based VMC assessment herein did not identify deficits at critical post-concussion time points. Therefore, other clinically translatable VMC assessments should be further investigated.

## 1. Introduction

Approximately 13-19% of all sports related injuries are concussions among American high school and collegiate athletes [1,2], causing a variety of somatic and psychological symptoms [3], as well as impairments in neuropsychological and cognitive function, and postural stability [3-5]. To evaluate a suspected concussion, the 5^th^ International Consensus Statement on Concussion in Sport (5^th^ CIS) recommends the use of a multifaceted assessment battery including self-reported symptoms, postural stability, and neurocognitive function [6]. This battery has an optimized sensitivity of 55% at 24-72 h post-injury, with clinical recovery on these tests typically occurring about 2 weeks post-injury [7,8]. However, these clinical tests are subject to practice effects secondary to repeat test administration, reducing sensitivity when assessing recovery [8,9]. Thus, it is not surprising that deficits are noted 30 days or more post-concussion when utilizing instrumented dual task gait, neuroimaging, or other laboratory measures [8,10-12]. This potential premature return to play (RTP) and lingering deficits could underlie the recently identified elevated rate of post-concussion subsequent musculoskeletal injury [13,14]. Therefore, a need exists for methods capable of identifying lingering deficits in recently concussed athletes.

Visual and oculomotor deficits, evidenced by increases in King-Devick test completion time or a positive vestibular/oculomotor screening, are becoming more commonplace post-concussion [15,16]. Moreover, concussion adversely affects visual motor coordination (VMC) up to 1 year post-injury which is well beyond the typical 2 weeks clinical recovery [17-19]. VMC engages visual perception to plan and control motor movements in response to a visual stimuli which involves multiple neural structures and pathways [20,21]. Specifically, visual stimuli information is filtered by the primary visual cortex, sent to the posterior parietal cortex, and used to help plan and control a motor response [20,22]. However, the posterior parietal cortex may be sensitive to the long-term effects of concussion, evidenced by residual deficits in upper limb visuomotor function (e.g., accuracy and movement velocity) up to 12-months following concussion [17,23]. In concussion management, neurocognitive tests broadly measure simple visual reaction time (SVRT), or visual motor processing speed, by assessing the speed one can press a key on a keyboard; a task that lacks ecological validity for athletes [24,25]. These instrumented assessments only measure SVRT and not VMC [24]. VMC is the ability to use visual information to initiate and guide limb movement, thus, these assessments may result in potential failure to identify deficits to track recovery [25]. Studies that identified upper limb VMC deficits utilized a computer with two color monitors and a steering wheel which has limited clinical feasibility due to the need for custom equipment and expert analysis [20,21]. Thus, a need exists for a laboratory based approach that can be easily interpreted by clinicians with increased ecological validity [17,23].

The Dynavision (D2 model, West Chester, OH) is a novel method for measuring VMC and has been used in collegiate student athletes to assess and manage concussion [26], study peripheral and central visual reaction times as a training device to improve VMC and eye function [26-28], and as an injury prevention tool [29,30]. The Dynavision A* and reaction time tests have been utilized as part of a vision training program suggesting a high degree of translation from the laboratory to the clinic and patient [26]. The A* test has been utilized by a collegiate football program as a form of visual motor skills training before and during the competitive season to improve performance, assess visuomotor reaction time, and reduce injury risk [29]. These players improved peripheral vision reaction time [30]; interestingly, individuals with slower visuomotor reaction time at baseline had higher rates of musculoskeletal injury [29]. Researchers posit that vision training improved field awareness and may possibly aide in preparatory awareness to reduce injury [30]. Thus, the Dynavision has the potential to be a highly effective and clinically useful measure of VMC.

Therefore, the purpose of this study was to determine if VMC, as assessed by Dynavision D2 A* and SVRT tests, would identify lingering deficits beyond clinical recovery in a concussed population of collegiate student-athletes. We hypothesized that the VMC tests would identify lingering deficits in all three Dynavision tasks, average A* score, reaction time (SVRT-RT), and movement time (SVRT-MT), at time point 1 (T1) but not at time point 2 (T2) (described in Procedures).

## 2. Methods

### 2.1. Participants

Participants consisted of 26 collegiate student-athletes from a NCAA Division I Football Championship Subdivision institution; 13 recently concussed (“concussion”) and 13 matched control participants (“control”) closely matched on sex, sport, age (within 3 years), and position (Table 1). The inclusion criteria for the concussion group were recent concussion diagnosed by the team physician consistent with the 4^th^ International Consensus Statement on Concussion in Sports (current statement in effect at the time of the study) (4^th^ CIS). The concussion participants had to complete the 6-day concussion RTP protocol (described in Procedures) within 14 days post-injury be considered a typically recovering concussion [3]. Concussion participants were excluded if they did not have a typically recovering concussion. Aside from the current concussion in the concussion group, the inclusion criteria for both groups were unrestricted participation as an intercollegiate student-athlete. Participants in either group were excluded if they had a current upper extremity injury, as identified on the self-reported health history survey, or self-reported any visual, vestibular, or neurological condition before concussion that would have interfered with performance. All participants provided written informed consent as approved by the university’s IRB.

**Table 1:** Participant demographics and anthropometrics.

	Number	Age (years)	Height (cm)	Weight (kg)	Concussion history	Days between T1 and T2	Days to clinical recovery
Concussion	13 (6 M)	18.8±1.1	174.7±12.2	75.5±23.2	0.38±0.77	22.8±3.5	6.6±3.8
Control	13 (6 M)	19.3±1.1	173.5±11.9	75.8±19.9	0.31±0.48	23.2±3.7	N/A
**Sport**	**Group**						

	**Concussion (*n*, &%)**				**Control(*n*, &%)**		

Cheer	5 (38.4)				5 (38.4)		
Football	4 (30.8)				4 (30.8)		
Women&’s basketball	1 (7.7)				1 (7.7)		
Men&’s soccer	1 (7.7)				1 (7.7)		
Volleyball	1 (7.7)				1 (7.7)		
Swim	1 (7.7)				1 (7.7)		

There were no significant differences (*P*>0.05) between groups for any of the demographic characteristics. M: Male. Anthropometric data are presented as Mean&±Standard deviation

### 2.2. Instrumentation

**Figure 1:**
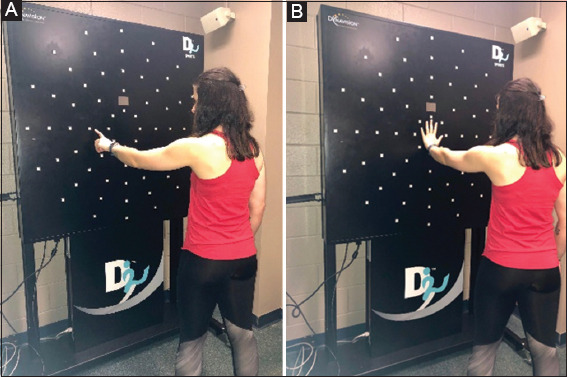
(A) Dynavision apparatus and participant set up for A* star test. (B) Dynavision apparatus and participant set up for SVRT test.

VMC was assessed with the Dynavision (D2 model, West Chester, Ohio) (Figure 1). Dynavision is a large black board with 64 lights organized into five concentric rings [31]. Two exams were used: (1) A* exam and the (2) SVRT test which assesses SVRT-RT and SVRT-MT. The A* exam assesses how quickly and accurately an individual can reach to touch visual stimuli. It has a test re-test reliability of 0.88, an ICC of 0.75 [31-33] and is moderately correlated with traditional VMC exams (0.42-0.75) [27]; however, its validity has not yet been studied. The second test, SVRT, does not have published reliability or validity, but has been utilized previously with concussed collegiate student-athletes [26].

The multifaceted concussion assessment battery consisted of the Immediate Post-concussion Assessment and Cognitive Testing (ImPACT), the Standardized Assessment of Concussion (SAC), Balance Error Scoring System (BESS), and the Graded Symptom Checklist (GSC) which are referred to as the “clinical assessments.” These assessments have been thoroughly described in the literature, are frequently utilized by clinicians, and deemed best practice by the 4^th^ CIS [3,34,35].

## 3. Procedures

At the beginning of their collegiate athletic career, all student-athletes performed the clinical assessment battery (i.e., baseline test) consisting of ImPACT, SAC, BESS, and GSC. During the academic year, concussions were identified by an athletic trainer and the diagnosis was confirmed by a licensed physician based on a clinical examination and supported by the assessment battery [3]. Following the diagnosis of a sports-related concussion, participants completed a standard post-concussion RTP protocol consistent with the recommendations of the 4^th^ CIS [3]. Briefly, concussed individuals were withheld from all activities for 24 to 48 h post-concussion for a period of reduced cognitive, physical, and social activities. Thereafter, concussion participants underwent serial administration of the clinical assessments until they matched or improved on their baseline values, self-reported asymptomatic, and received clearance from the team physician indicating “clinical recovery.” Following clinical recovery, participants completed a 6-day RTP progression also consistent with the 4^th^ CIS recommendations and received final medical clearance from the team physician [3]. Any participants who experienced a reemergence of symptoms during the progressive exercise protocol were excluded from the study.

Concussion group participants were assessed at two time points: Less than 48 h of clinical recovery (T1) and 30 days post-concussion (T2). The matched control participants were recruited and enrolled on a one to one basis to the concussion participant after the concussion participant completed the last assessment. The control participants completed two test sessions matching “clinical recovery” and “30 days” and the time between assessment was consistent (±1 day) with the concussion participant (Table 1).

Dynavision testing consisted of two assessments: The A* exam and SVRT test. For the A* exam, participants stood within an arm’s reach of all lights on the apparatus (Figure 1A).

Participants were instructed to hit illuminated lights, deactivating them, using either hand as fast as possible for 60-s. Following an established 30-s warm up protocol in which participants performed the A* exam until they no longer improved (to reduce a practice effect), participants completed five trials of the A* exam [27,31]; the test outcome measure was the A* average score or the mean number of lights deactivated in 60-s across the five trials [26].

For the SVRT test, participants held down a button on the center of the board, during which a 2^nd^ button 30 cm away would light up, they then released the original button and reached to touch the 2^nd^ button as quickly as possible with the same hand (Figure 1B). Three warm up trials were completed, then five recorded trials on each hand. There are two measures for the SVRT: SVRT-RT and SVRT-MT.

SVRT-RT is the time required for the participant to perceive the light and lift their hand from the starting button and the SVRT-MT is the time between releasing the original button and pressing the target light. Both the SVRT-RT and SVRT-MT times were averaged across the five trials, and then the mean score between the two hands was calculated for each session to give a single score for each participant.

### 3.1. Statistical analysis

Descriptive statistics for demographics and dependent variables were calculated. An independent samples t-test was used to assess group demographic differences. A 2 (group) × 2 (session) repeated measures ANOVA was used to compare each of the three dependent variables (A* average score, SVRT-RT, and SVRT-MT) along with effect sizes for the interaction (ƞ^2^) and differences in mean scores (Cohen’s *d*). As no pre-injury measures were available for Dynavision assessments, exploratory Tukey *post hoc* tests were conducted to investigate group differences at each time point. Normality of the data was checked using the Kolmogorov–Smirnov tests and all dependent variables were normally distributed. All statistical analyses were performed on SPSS v. 26 (SPSS Inc., Chicago, IL) and alpha levels were set a priori at 0.05.

## 4. Results

All 26 participants completed the A* exam. One control participant did not complete the SVRT due to technical problems with the Dynavision; therefore, the SRVT measures represent 24 participants (12 concussion and 12 control).

There were no significant interactions for A* Average Score (*F*(1,24)=0.036, *P*=0.849, ƞ^2^=0.013, *post hoc* observed power = 0.054), SVRT-RT (*F*(1,22)=0.319, *P*=0.575, ƞ^2^=0.025, *post hoc* observed power = 0.086), or SVRT-MT (*F*(1,22)=1.179, *P*=0.188, ƞ^2^=0.030, *post hoc* observed power = 0.258).

**Figure 2:**
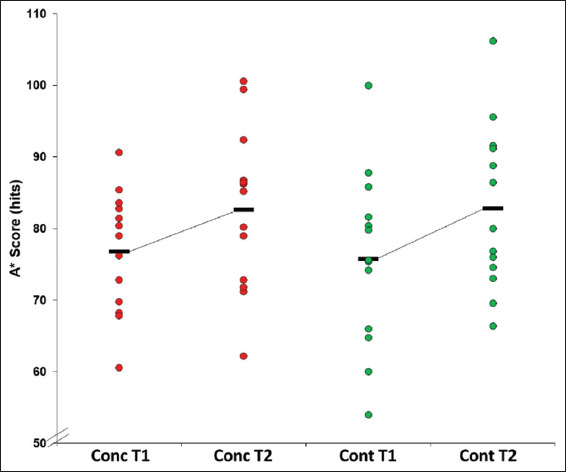
Mean A* score by time point by group.

There was a significant main effect for time (*F*(1,24)=38.1, *P*<0.001, ƞ^2^=0.61) for the A* average score with both groups improving their score between sessions (T1: 76.3±10.4 hits; T2: 82.7±11.2 hits) (Figure 2).

Mean A* Score: Concussion T1: 76.8±8.5; concussion T2: 82.6±10.9* (*d* = 0.59); control T1: 75.8±12.4; control T2: 82.7±11.6* (*d* = 0.57). *Main effect for time, p<0.001.

**Figure 3:**
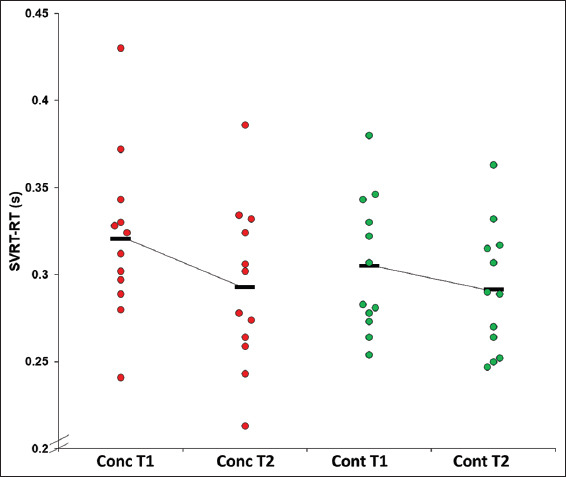
Mean SVRT-RT by time point by group.

There was a significant main effect for the time for SVRT-RT (*F*(1,22)=4.9, *P*=0.039, ƞ^2^=0.18) with both groups improving their time (faster) between sessions (T1: 0.31±0.04; T2: 0.29±0.04 s) (Figure 3).

Mean SVRT-RT for the concussion group was 0.32±0.05s at T1 and 0.29±0.05s* at T2 (*d* = 0.60). The control group had a mean SVRT-RT of 0.30±0.04s at T1 and T2: 0.29±0.04s* at T2 (*d* = 0.25). *Main effect for time, *P*<0.05.

**Figure 4:**
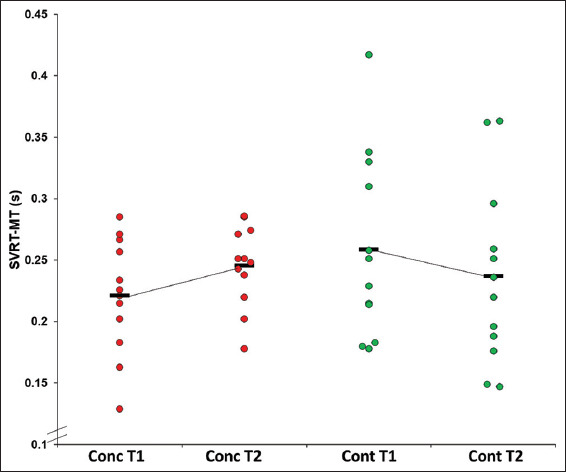
Mean SVRT-MT by time point by group.

There were no significant main effects for time for SVRT-MT (*F*(1,22)=0.007, *P*=0.933, ƞ^2^=0.008) (Figure 4).

Mean SVRT-MT for the concussion group was 0.22±0.09s at T1 and 0.25±0.03s at T2 (*d* = −0.44). Mean SVRT-MT for the control group was 0.26±0.08s at T1 and 0.24±0.06s at T2 (*d* = 0.28).

There were no main effects for Group for the A* average score (*F*(1,24) = 0.020, *P*=0.889, ƞ^2^<0.001), SVRT-RT (*F*(1,22)=0.479, *P*=0.493, ƞ^2^=0.015), or SVRT-MT (*F*(1,22)=0.700, *P*=0.407, ƞ^2^=0.003). There were no differences between groups at T1 for the A* average score (*P*=0.816), SVRT-RT (*P*=0.379), or SVRT-MT (*P*=0.131). Furthermore, there were no group differences at T2 for the A* average score (*P*=0.972), SVRT-RT (*P*=0.928), or SVRT-MT (*P*=0.725).

## 5. Discussion

The purpose of this study was to determine if VMC tests, through the use of the Dynavision, could provide a translational approach to identify persistent deficits across clinical landmarks of concussion recovery. Despite emerging evidence of persistent neurological deficits following clinical recovery [8,12], there were no VMC deficits identified herein. However, a practice effect appears to exist as there were significant improvements in both A* average score and SVRT-RT over the two sessions despite participants completing the manufacturer recommended warm-up designed to reduce the practice effect. These results suggest that either there are no lingering deficits in VMC post-concussion in this population or the Dynavision was not sensitive enough to identify the lingering deficits.

Contrary to our results showing no deficits, Heitger *et al*. found persistent deficits for a year post-concussion when assessing VMC by tracking tasks (e.g., on a computer screen) using a steering wheel in symptomatic mild closed head injury patients (mean age: 29.1±12.7 years) with Glasgow Coma Scale scores between 13 and 15 following an initial visit to an emergency department [17]. In addition, VMC dysfunction, evidenced by slowed central and peripheral visual reaction times, assessed by Dynavision has been found post-concussion in collegiate student athletes with visual dysfunction [26]. However, the participants in our study were asymptomatic, post-concussion, and denied visual dysfunction which may explain the difference in results [26]. Herein, there were no significant interactions for A* average score, SVRT-RT, or SVRT-MT. In addition, there were no differences between groups on any test at either T1 or T2 when assessed by the Dynavision. The participants in this study were asymptomatic within 14 days (6.46±3.41 days), collegiate student-athletes, and did not warrant an emergency department visit, and therefore likely represents a different post-concussion population than Heitger’s participants that were symptomatic and older [17]. In addition, our participants did not have physician diagnosed post-concussion visual dysfunction based on visual symptom reporting like the student athlete participants in Clark’s study which may be why the Dynavision was unable to identify VMC deficits in this population [26]. As such, Dynavision may not be a sensitive enough measure to assess VMC in typically recovering concussed athletes without visual dysfunction.

Hick’s law suggests that as the possible responses in a reaction time test increases, the reaction time will increase as well [28]. This indicates an increased load on the central nervous system’s planning and initiation of a motor response [18,36]. We hypothesized that the A* exam, which utilizes 64 lights at five distances and 16 angles from the board’s center, would provide a sufficiently challenging test of VMC to identify lingering deficits post-concussion; however, our results revealed no deficits in VMC in the concussed group. These results suggest that either VMC is recovered by the symptom-free time point, or that the A* test is not a sensitive enough instrument to capture these deficits. Simple reaction time is commonly used as a component for neuropsychological testing, such as ImPACT, in concussed student-athletes, and has shown that visual reaction time recovers within 2 weeks post-injury [24,25]. Herein, these athletes’ SVRT would have recovered by T1 since return to baseline score on ImPACT was part of the clinical recovery criteria. As such, this is most likely the reason there were no group differences in VMC task outcomes when assessed by Dynavision at either time point.

There was significant improvement in the score of both groups across the two sessions for mean A* score and the SVRT, indicating a potential learning effect from repeat test administration. It should be noted that Dynavision was created as a psychomotor training tool and, despite completing the prescribed warm-up designed to mitigate the practice effect, the participants continued to improve suggesting that three warm up trials may not be sufficient [31,37]. When using the Dynavision SVRT protocols, it should be noted that response time is the summation of reaction time and movement time [18,36,38]. Reaction time reflects the temporal delay required for the CNS to recognize a stimulus and initiates a motor response [18,36,38]. Movement time is a reflection of the time required for the peripheral nervous system to recruit the appropriate motor units to complete the task [38,39]. The improvement in SVRT-RT and A* average score for both groups may have indicated an increased speed in the CNS planning the direction and magnitude of arm movement in response to the stimulus as a result of repeat exposure to the same task [18,38,40]. Furthermore, the SVRT-RT test re-test reliability has not been established and the participants herein demonstrated significant improvements between test sessions with moderate effect sizes (*d*=0.59 and *d*=0.57 in the concussion and control groups) in A* average score despite the approximately 3-week test interval. This practice effect reduces the clinical and translational utilization of the A* exam to identify persistent deficits in VMC in athletes post-concussion; instead, it may have assessed how quickly one could adapt to the novel motor task. A revised testing protocol would need to be developed that would reduce the practice effect to better assess the best performance of the participants’ VMC. However, this seems difficult as Dynavision A* exam has been shown to improve visuomotor responsiveness through training, so practice effects may be unavoidable [29]. However, even if practice effects are unavoidable, if there were post-concussion deficits in VMC, we would expect limited improvement in the concussion group as compared to the control group; as this did not occur we can infer than the concussion group did not have deficits in VMC.

The A* exam has been utilized by Wilkerson *et al*. to train visuomotor reaction time in athletes [29]. Interestingly, they reported a pre-season baseline median score of 85 hits, greater than the median score (Concussion: 76.2 hits; Control: 77.7 hits), and mean scores (Concussion: 76.8±8.5 hits; and Control: 75.8±12.4 hits) in both of our groups at T1. Arbitrarily and coincidentally, this median score cut point of <85 hits provided discrimination between injured and uninjured players. Hence, while the Dynavision A* exam may not identify VMC deficits, it may be useful as a tool for identify injury risk [29]. Unfortunately, the design of this study precluded the inclusion of baseline performance and therefore no conclusions can be drawn related to Dynavision’s predictive capabilities for concussion.

Laboratory assessments of VMC, while limited by costs and equipment, can provide critical basic scientific knowledge to elucidate neurological impairments following concussions such as discriminating between reaction and movement time [28,29]. However, a need still exists of a clinically feasible alternative to bridge the translational gap between instrumented and clinical reaction times (CRT). One method may be the CRT test, a component of the Concussion Assessment, Research and Education (CARE) consortium protocol which studies the natural history of neurobiological and clinical recovery in student-athletes and military cadets [35]. This is a visuomotor test requiring the subject to catch a falling object with their hand [35]. The CRT has comparable test characteristics to other reaction time assessment tools (i.e., ImPACT), showing deficits in athletes post-concussion with 75% sensitivity and 68% specificity for concussion [41]. Data from the CARE consortium revealed a year 1 to year 2 test re-test reliability off 0.32 (0.21-0.43) with a small effect size (Cohen’s *d* = 0.34) [34]. When compared to computerized reaction time measures, the CRT showed favorable test re-test reliability (ICC = 0.645 for CRT and ICC = 0.512 for computerized CogState Sport) in 102 NCAA Division I athletes [42]. Future studies should continue to investigate translational approaches to VMC through direct comparison between laboratory and clinical assessments to optimize the test battery.

This study was limited by the lack of healthy pre-injury data and therefore within-subjects healthy versus post-concussion performance could not be investigated. An important limitation is that the results of the study were underpowered for the interactions (*post hoc* observed power was .054 (A* Average Score), 086 (SVRT-RT), and 0.258 (SVRT-MT). However, we attempted to recruit all concussed student-athletes at one institution over the course of an academic year, but were limited by exclusion criteria and limited number of individuals willing to participate. This is particularly noteworthy in the SVRT-MT outcome where the concussion participants got worse (11.09%) while the control participants got better (8.38%), but the observed power was low (0.258). A power analysis indicated that 64 subjects per group would have been required to be adequately powered for SVRT-MT measures. Subjectively, some participants appeared highly competitive and motivated to excel during the assessments and therefore motivational status could limit the results, especially when expanding on pre-college career baseline assessments.

## 6. Conclusions

There were no group differences between control and recently concussed participants on two VMC tests assessed by Dynavision suggesting either the assessment may lack sensitivity to assess VMC in recently concussed collegiate athletes following clinical recovery or the student athletes may not have VMC deficits at RTP. In addition, there was a significant practice effect for both the A* and SVRT-RT outcomes which limits its clinical utility for follow-up assessments and tracking recovery in collegiate student athletes.

### Relevance for Patients

The need for highly sensitive and specific assessments of concussion recovery remains an ongoing challenge to the sports medicine community [8,12]. Specifically, a VMC assessment with high efficacy could improve concussions management through more accurate determination of physiological recovery. This improved concussion management could help reduce the risk of subsequent concussions and musculoskeletal injuries thus allowing for better patient outcomes and continued performance of their activities of daily living [6,13,14].

### Conflicts of Interest Statement

The authors have no conflicts of interest to report.

### Funding

This project was funded, in part, by a grant from NIH/NINDS: 1R15NS070744-01A1.
